# Sténose vaginale acquise: à propos d’un cas et revue de littérature

**DOI:** 10.11604/pamj.2019.34.175.15036

**Published:** 2019-12-04

**Authors:** Yousra Krimou, Ahmed Guennoun, Nisrine Mamouni, Sanae Erraghay, Chahrazed Bouchikhi, Abdelaziz Banani

**Affiliations:** 1 Department of Obstetrics and Gynecology I, University Hospital Center Hassan II, Fez, Morocco

**Keywords:** Sténose acquise, post partum, aménorrhée, dilatation, libération, Acquired stenosis, postpartum, amenorrhea, dilatation, release

## Abstract

La sténose vaginale ou gynatrésie est une pathologie congénitale et fait partie du syndrome de Mayer-Rokitansky-Kustner-Hauser. La sténose vaginale acquise est une complication rare de l'accouchement par voie vaginale et peut résulter d'une infection, de charlatans ou d'une blessure à la naissance ou d'une hypo-œstrogénie du post-partum. Nous rapportons le cas de sténose vaginale totale post-partum secondaire à la négligence des lésions vaginales. Une patiente de 19 ans, qui a accouché spontanément à domicile un mort-né, a consulté pour une aménorrhée secondaire de deux ans associée à des douleurs pelviennes chroniques. La patiente rapporte la survenue de multiples déchirures vaginales non suturées. L'IRM pelvienne montre une sténose vaginale complète étendue à environ 25mm avec une rétention hématologique en amont et un hématosalpinx bilatéral. Patiente a bénéficié d'une libération des adhérences vaginales suivie d'une dilatation vaginale régulière. Seuls deux cas ont été signalés dans la littérature. La douleur et la dyspareunie étaient les symptômes les plus courants. Tous les cas ont été traités par une libération des synéchies et une dilatation vaginale.

## Abstract (en)

Vaginal stenosis or gynatresia is a congenital disease and it is part of Mayer-Rokitansky-Kustner-Hauser syndrome. Acquired vaginal stenosis is a rare complication of vaginal delivery and may be caused by an infection, charlatans, birth injury or postpartum hypoestrogeny. We report a case of postpartum complete vaginal stenosis secondary to vaginal injuries as a result of medical negligence. The study involved a 19 year old patient, who had had dead child born vaginally at home, presenting with a two-year history of secondary amenorrhea associated with chronic pelvic pain. The patient reported the occurrence of multiple not sutured vaginal tears. Pelvic MRI showed complete extended vaginal stenosis of about 25mm with upstream haematological retention and bilateral hematosalpinx. The patient underwent release of vaginal adhesions followed by regular vaginal dilation. Only two cases have been reported in the literature. Pain and dyspareunia were the most common symptoms. All cases were treated by a release of the synechias and vaginal dilation.

## Introduction

Les rétrécissements acquis du vagin constituent une situation exceptionnelle qui est peu intégrée dans l’enseignement actuel. Nunns *et al.* [1] la décrivent comme l’impossibilité d´insérer deux doigts dans le vagin. Flay et Matthews [2] la définissent comme le raccourcissement du vagin à moins de 8cm. Bruner *et al.* [3] l'identifient comme étant une diminution de la longueur vaginale par rapport à la normale de 8-9cm tandis que Schover *et al.* [4] la classent en termes de muqueuse et de capacité vaginale en normale, légèrement modifiée, ou gravement changée. Hartman et Diddle [5] classent les changements sténotiques numériquement de un à trois. Un score de niveau 1 représente une absence de sténose, le niveau 2 est lié à la sténose du tiers supérieur du vagin et le niveau 3 indique la présence de sténose atteignant plus que le tiers supérieur. Les étiologies sont diverses et fonction de l'âge: Séquelle d'une dermatose ou d'une thérapeutique chez les femmes âgées, ou de mutilation génitales chez les jeunes filles ou traumatique. Nous rapportons le cas de sténose vaginale totale qui a survenue post-partum secondaire à la négligence des lésions vaginales.

## Patient et observation

**Figure 1:**
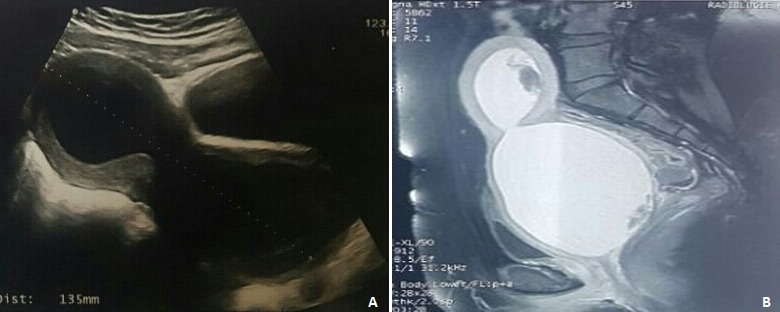
(A,B) hématocolpos et hématométrie en amont d’une sténose vaginale complète

Mme A.M âgée de 19 ans, primipare, sans antécédents pathologiques notables, s'est présentée dans notre formation pour la prise en charge d'une aménorrhée secondaire de deux ans associée à des douleurs pelviennes chroniques type pesanteur. Selon la patiente, elle a accouché il y a trois ans par voie vaginale un mort-né prématuré après un long travail à domicile sans assistance médicale. Elle a également décrit la survenue de plusieurs déchirures vaginales non suturées. Après l´accouchement, elle a accusé une gêne vaginale, une oligoménorhée mais les cycles étaient réguliers. À l’examen, On a découvert une sténose vaginale complète niveau 3 selon la classification de Hartman. L'échographie pelvienne a montré un énorme hématocolpos mesurant 84*58mm associé une hématométrie (Figure 1 A). L’IRM pelvienne a mis en évidence une sténose vaginale inférieure complète étendue sur 25mm avec une importante rétention hématique en amont et un hématosalpinx bilatéral (Figure 1 B). La patiente a bénéficié d'une dissection de proche en proche des adhérences vaginales jusqu'à visualisation du col utérin avec une dilatation à la bougie qui a été maintenue par la mise en place de préservatif pendant 24 heures au début puis uniquement pendant les nuits pendant 1 semaine.

## Discussion

La sténose vaginale est souvent congénitale et survient dans le cadre du syndrome de Mayer-Rokitansky-Kustner-Hauser qui a une incidence de 4 000-10 000 naissances [6]. La sténose vaginale acquise ou la gynatrésie acquise est commune dans les pays en voie de développement en particulier le Nigeria où l’incidence est d´environ 7/1000 femmes vu la fréquence des mutilations génitales féminines. On peut distinguer comme causes acquises de sténose du vagin deux grands groupes étiologiques: les dermatoses (lichen scléreux vulvaire [7], lichen plan, etc.), les conséquences d'une thérapeutique le plus souvent après la radiothérapie ou une réaction du greffon contre l'hôte [8] soit dans le cadre du syndrome de Steven-Johnson ou après une nécrolyse épidermique toxique. La gynatrésie dans les pays en voie de développement est surtout causée par les traumatismes vaginaux obstétricaux, les mutilations génitales féminines et l’insertion de pessaires caustiques en intra vaginal pour traiter les fibromes, les prolapsus ou interrompre une grossesse non désirée. Seuls deux cas de sténose vaginale survenue en post-partum ont été rapportés dans la littérature. Shipra *et al*. [9] a rapporté un cas de sténose vaginale secondaire à l'oubli de compresse en intra vaginal après un accouchement par voie basse. Howard *et al*. [10] ont rapporté un cas d’agglutination vaginale dû à une hypo oestrogénie sévère observée au cours de l'allaitement maternel. Cependant, dans notre cas, la sténose vaginale est due aux déchirures vaginales non suturées. Dans le cas de Shipra *et al*. la patiente a consulté pour la prise en charge d'une infertilité secondaire, une dyspareunie et une dysménorrhée, les cycles étaient réguliers. Dans le cas de Howard *et al.* La sténose vaginale a été découverte fortuitement lors d'une consultation de routine 8 semaines après l'accouchement. Dans notre cas, la patiente a accusé une aménorrhée de deux ans. Aucune patiente ne présentait un hématocolpos ou une hématométrie. Toutes les patientes ont bénéficié d'une dissection des adhérences vaginales suivie d’une dilatation vaginale régulière pour éviter la récidive et pour étirer la muqueuse vaginale. Toutes les patientes ont rapporté des rapports sexuels satisfaisants ainsi la disparition totale de tous les symptômes.

## Conclusion

La sténose vaginale acquise est une complication rare de l'accouchement vaginal. Les étiologies sont diverses mais le traitement est simple et repose sur la libération des adhérences ainsi que la dilatation vaginale.

## Conflits d’intérêts

Les auteurs ne déclarent aucun conflit d’intérêts.
